# Beneficial Effects of Mineralocorticoid Receptor Pathway Blockade against Endothelial Inflammation Induced by SARS-CoV-2 Spike Protein

**DOI:** 10.3390/biomedicines9060639

**Published:** 2021-06-03

**Authors:** Eva Jover, Lara Matilla, Mattie Garaikoetxea, Amaya Fernández-Celis, Pieter Muntendam, Frédéric Jaisser, Patrick Rossignol, Natalia López-Andrés

**Affiliations:** 1 Cardiovascular Translational Research, Navarrabiomed (Miguel Servet Foundation), Instituto de Investigación Sanitaria de Navarra (IdiSNA), Complejo Hospitalario de Navarra (CHN), Universidad Pública de Navarra (UPNA), 31008 Pamplona, Spain; eva.jover.garcia@navarra.es (E.J.); lara.matilla.cuenca@navarra.es (L.M.); mattiegaraiko@gmail.com (M.G.); amaya.fernandez.decelis@navarra.es (A.F.-C.); 2 G3 Pharmaceuticals, Burlington, MA 01803, USA; Pmuntendam@g3pharma.com; 3 Centre de Recherche des Cordeliers, INSERM, Sorbonne Université, USPC, 75006 Paris, France; frederic.jaisser@inserm.fr; 4 Centre d’Investigations Cliniques-Plurithématique (INSERM CIC-PT 1433), UMR 1116, CHRU, Université de Lorraine, 54500 Vandoeuvre-Les-Nancy, France; p.rossignol@chru-nancy.fr; 5 French-Clinical Research Infrastructure Network (F-CRIN) Cardiovascular and Renal Clinical Trialists (INI-CRCT), 54500 Nancy, France

**Keywords:** SARS-CoV2, Spike, endothelial cells, inflammation, mineralocorticoid receptor

## Abstract

Background: Vascular endothelial cells activation and dysfunction mediate inflammation and abnormal coagulation in COVID-19 patients. Mineralocorticoid receptor (MR) signaling and its downstream target Galectin-3 (Gal-3) are known to mediate cardiovascular inflammation and might be involved in the pathogenesis of COVID-19 complications. Accordingly, we aimed to investigate the potential beneficial effects of MR antagonism and Gal-3 inhibition on the inflammatory response induced by SARS-CoV-2 Spike protein in human aortic endothelial cells (HAECs). Methods: HAECs were treated with recombinant SARS-COV2 Spike (S) protein. MR antagonists (namely spironolactone and eplerenone) or the Gal-3 inhibitor G3P-01 were supplemented before and after S protein challenge. HAECs supernatants were assessed by ELISA or Western blotting. Results: HAECs treated with recombinant S protein resulted in enhanced secretion of inflammatory molecules (interleukin-6, monocyte chemoattractant protein-1, interleukin-18, interleukin-27, and interferon-γ) as well as in the thrombosis marker plasminogen activator inhibitor (PAI)-1. This was prevented and reversed by both MR antagonists and G3P-01. Conclusions: These findings indicate that MR/Gal-3 pathway blockade could be a promising option to reduce endothelial inflammation in SARS-CoV-2 infection.

## 1. Introduction

The onset of severe COVID-19 presentation and subsequent complications encompasses respiratory symptoms, subsequently associated with a cytokine storm, which may combine disseminated intravascular coagulation and multiple organ failure. SARS-CoV-2 binds to the host cell receptor Angiotensin-converting enzyme 2 (ACE2) through the Spike glycoprotein, which mediates membrane fusion and virus entry. The S protein is then cleaved into the N-terminal S1 subunit and C terminal S2 subunit by the host transmembrane protease serine 2 (TMPRSS2) [1].

Both ACE2 and TMPRSS2 are expressed by endothelial cells [2]. It has been recently proposed that COVID-19 is an endothelial disease [3,4]. Epidemiological studies have reported that severe COVID-19 clinical history is often complicated by ageing and clinical conditions such as hypertension, diabetes, obesity, cardiovascular disease, and exposure to androgen hormones [5]. These so-called ‘COVID-19 risk factors’ are per se associated with underlying endothelial damage and dysfunction, leading to chronic systemic inflammation and hypercoagulation. There is therefore an urgent need to identify effective treatment options bearing direct or indirect endothelial protection [6].

Mineralocorticoid receptor antagonists (MRAs), namely Spironolactone and Eplerenone, may prevent acute lung injury in COVID-19 infection and provide additional protection for patients at highest risk of severe pneumonia towards their pleiotropic effects [7]. Spironolactone is an interesting candidate to treat COVID-19 symptoms [8]. Studies performed in COVID-19 patients have reported an increase in inflammatory cytokines (cytokine storm) including interleukin (IL)-6, IL-10, IL-18, interferon (INF) γ, or monocyte chemoattractant protein-1 (CCL-2) [9], IL-27 [10], as well as in the thrombosis marker plasminogen activator inhibitor (PAI)-1 [11]. Of interest, COVID-19 patients also exhibit enhanced levels of the MR-regulated proinflammatory molecule Galectin-3 (Gal-3) [12]. Mineralocorticoid receptor (MR) activation plays a prominent role in cardiovascular inflammation [13,14,15]. Moreover, Gal-3 enhances inflammation and fibrosis in many pathophysiological contexts, including atherosclerosis or cardio-renal diseases [15,16,17,18,19] and plays a critical role in venous thrombosis towards IL-6 dependent mechanisms [20]. Importantly, SARS-Cov-2 receptor binding domain shares sequence similarity with galectins, which in turn are known to bind blood group antigens [21]. A recent study suggested that galectins might contribute to the stability and preference infectability of SARS-CoV-2 on blood group A respiratory epithelium, thus striking the COVID-19 progression [22]. Interestingly, an association between MR pathway and Gal-3 has been established. MR mediated the increase in Gal-3 expression in a dose- and time-dependent manner in vascular cells, emerging Gal-3 as a necessary factor allowing MR-induced vascular, cardiac, and renal inflammation [15,17,18,19]. Thus, both MR and Gal-3 inhibition have been proven beneficial in cardiovascular pathologies including heart failure and hypertension [15,17].

Our study was designed to understand the possible beneficial effects of MR pathway blockade against SARS-CoV-2 effects on endothelial inflammation.

## 2. Materials and Methods

Major resources are listed in Table 1.

### 2.1. Cell Culture and Treatments

Human aortic endothelial cells (HAECs) from 4 different male donors were obtained from Promocell. HAECs were grown in endothelial cell growth media following the manufacturer’s instructions (Promocell). All assays were done at 37 °C, 95% sterile air, and 5% CO_2_ in a saturation-humidified incubator. Cells were used between passages 3–5.

HAECs were treated with recombinant SARS-CoV2 Spike protein (1 μg/mL, R&D Systems) for 24 to 72 h for protein studies. The duration and the dose of the treatment was chosen based on previous studies (data not shown). Cells were treated with Spironolactone (10^−6^ M, Sigma Aldrich), Eplerenone (10^−5^ M, Sigma Aldrich), or the Galectin-3 inhibitor G3P-01 (1 mg/mL, G3 Pharmaceuticals) 1 h before Spike stimulation to assess the preventive effect of the aforementioned MRAs and the Gal-3 inhibitor. Cell supernatants were collected at 24, 48, and 72 h. In another set of experiments, HAECs were treated with recombinant Spike (1 μg/mL) for 24 h and then co-treated with Spironolactone (10^−6^ M), Eplerenone (10^−5^ M), or G3P-01 (1 mg/mL) for further 24 and 48 h to study if MR and Gal-3 antagonism can revert the pathological effect of SARS-CoV2 Spike protein on HAECs.

### 2.2. Western Blot Analysis

Aliquots of 10 µg of total proteins were prepared from cells, electrophoresed on SDS polyacrylamide gels and transferred to Hybond-c Extra nitrocellulose membranes (Bio-Rad). Membranes were incubated with primary antibodies for: ACE2 (1:50, Abcam), TMPRSS2 (1:100, Santa Cruz), or β-actin (1:100, Santa Cruz). Stain free detection was also used as loading control. After washing, detection was made through incubation with peroxidase-conjugated secondary antibody, and developed using an ECL chemiluminescence kit (Amersham). After densitometric analyses, optical density values were expressed as arbitrary units. All Western blots were performed at least in triplicate for each experimental condition.

### 2.3. Cytokine Array

After 24 h incubation, cell supernatants were collected and analyzed using a human cytokine array kit following manufacturer’s instructions (R&D Systems). A pool of at least 12 supernatants per condition of 3 independent experiments were used. The results were normalized to the control condition in each cell type. Data were expressed as a fold change relative to controls.

### 2.4. Enzyme-Linked Immuno Sorbent Assay (ELISA)

Secretion of IL-6, CCL-2, IL-18, IL-27, IFN-γ, PAI-1, and Gal-3 was assessed in cell supernatants by ELISA according to the manufacturer’s instructions (R&D Systems).

### 2.5. Statistical Analyses

Continuous variables are shown as mean ± SEM. Normal distribution was verified by means of the Kolmogorov–Smirnov test. Normally distributed data were compared using a one-way analysis of variance (ANOVA) test followed by a Dunnett’s multiple comparison post-hoc analysis. Non-parametric data were analyzed using the Kruskal Wallis test followed by Mann Whitney U test. Statistical significance was accepted at *p* < 0.05. Analyses were performed using GraphPad Prism 5.0 (GraphPad Software Inc). 

## 3. Results

### 3.1. SARS-CoV-2 Spike Protein Effects on HAECs

**Figure 1:**
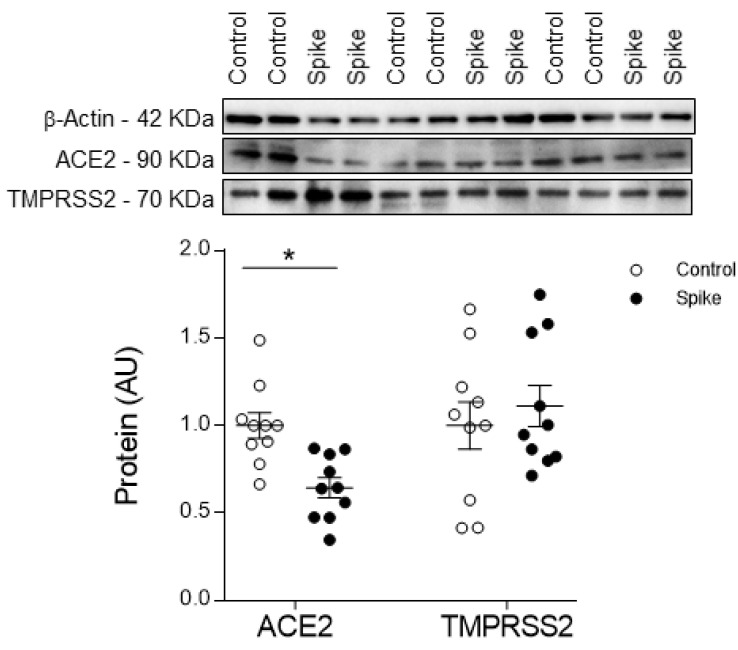
ACE2 and TMPRSS2 expression in HAECs. Quantification of ACE2 and TMPRSS2 in controls and Spike-treated cells. Whole original blots are displayed for ACE2, TMPRSS, and β-actin. The graphs represent the mean ± SEM of each group in arbitrary units (AU) normalized to the signal of stain-free protein gels or β-actin. *n* = 10 wells per condition from 4 independent HAECs donors. * *p* < 0.05 vs. control.

The expression of ACE2 and TMPRSS2 was confirmed in HAECs (Figure 1). Stimulation with SARS-CoV-2 Spike protein for 24 h decreased (36%, *p* = 0.0012) ACE2 expression and did not modify TMPRSS2 expression (Figure 1).

**Figure 2:**
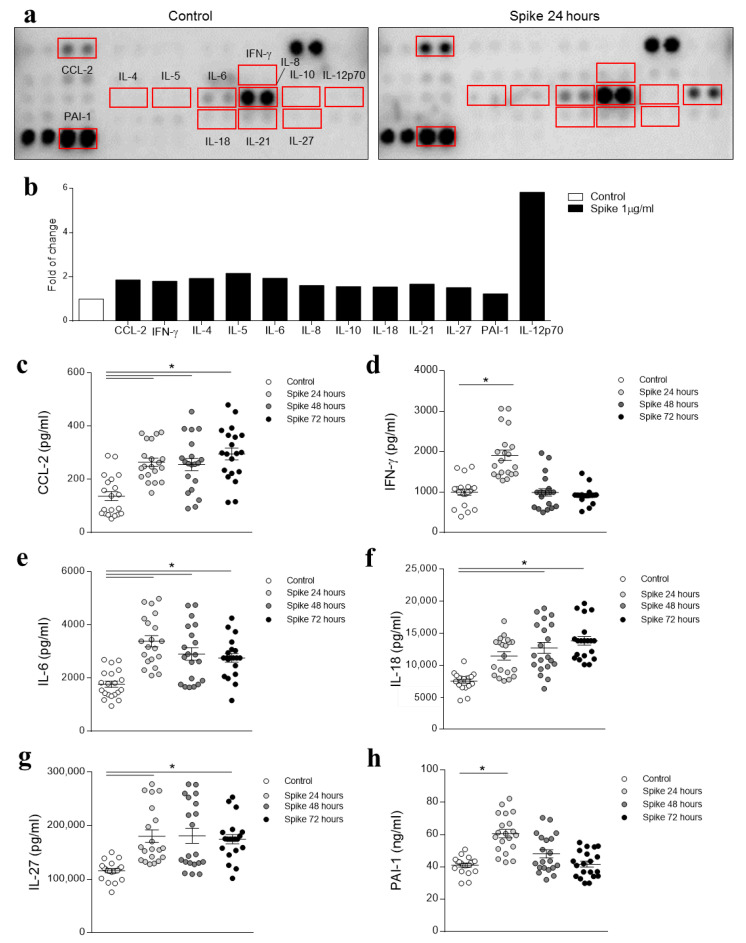
Effects of recombinant Spike protein on HAECs. Representative images of human cytokine array blots probed with the supernatant samples of controls and Spike-treated cells for 24 h (*n* = 12 supernatants per condition of 3 independent experiments). Each blot represents the immunoreactive staining with each cytokine. The lack of dots represented the negative and blank control. The blots marked inside the box are those selected (**a**). The fold-change of cytokines was determined by comparing the pixel intensity of the respective blots to that of the positive control on the same array (**b**). Quantification of CCL-2, IFN-γ, IL-6, IL-18, IL-27, and PAI-1 secretion in HAECs treated with recombinant Spike for 24, 48, and 72 h (Control, *n* = 20; Spike 24 h, *n* = 20; Spike 48 h, *n* = 20; Spike 72 h, *n* = 20) (**c**–**h**). Results were analyzed using one-way-ANOVA followed by Dunnet’s post-hoc multiple comparisons tests. * vs. control.

The effects of recombinant SARS-CoV-2 Spike protein on HAECs were analyzed and quantified using a cytokine array (Figure 2a). Those molecules increased by the Spike protein treatment for more than 50% have been selected. Thus, CCL-2, IFN-γ, IL-4, IL-5, IL-6, IL-8, IL-10, IL-18, IL-21, IL-27, PAI-1, and IL-12p70 cytokines were validated by ELISA. The increase of cytokines in recombinant Spike-treated HAECs for 24 h relative to controls were: 1.86-fold for CCL-2; 1.80-fold for IFN-γ; 1.93-fold for IL-4; 2.16-fold for IL-5; 1.94-fold for IL-6; 1.61-fold for IL-8; 1.56-fold for IL-10; 1.54-fold for IL-18; 1.67-fold for IL-21; 1.51-fold for IL-27; 1.23-fold for PAI-1; 5.83-fold for IL-12p70 (Figure 2b). Based on previous studies showing their involvement in COVID-19 infection, CCL-2, IFN-γ, IL-6, IL-18, IL-27, and PAI-1 were further studied in additional experiments at 24, 48, and 72 h. Treatment with recombinant SARS-CoV-2 Spike protein enhanced CCL-2 secretion was increased in a time-dependent manner by recombinant Spike (1.94-fold, *p* = 0.0012 for 24 h; 1.88-fold, *p* = 0.0018 for 48 h; and 2.17-fold, *p* < 0.0001 for 72 h) (Figure 2c). IFN-γ secretion was only increased after 24 h of Spike protein treatment (1.92-fold, *p* = 0.0002) (Figure 2d). Recombinant Spike increased IL-6 secretion at 24 (1.92-fold, *p* < 0.0001), 48 (1.64-fold, *p* = 0.0003), and 72 (1.56-fold, *p* = 0.0018) hours (Figure 2e). IL-18 secretion was time-dependently enhanced by Spike stimulation (1.53-fold, *p* = 0.001 for 24 h; 1.69-fold, *p* < 0.0001 for 48 h; and 1.84-fold, *p* < 0.0001 for 72 h) (Figure 2f). Treatment with recombinant Spike protein augmented IL-27 secretion at 24 (1.56-fold, *p* < 0.0001), 48 (1.56-fold, *p* = 0.0003), and 72 (1.51-fold, *p* < 0.0001) hours (Figure 2g). Finally, treatment with recombinant Spike increased the secretion of the thrombotic marker PAI-1 only at 24 h (1.37-fold, *p* < 0.0001) (Figure 2h).

**Figure 3:**
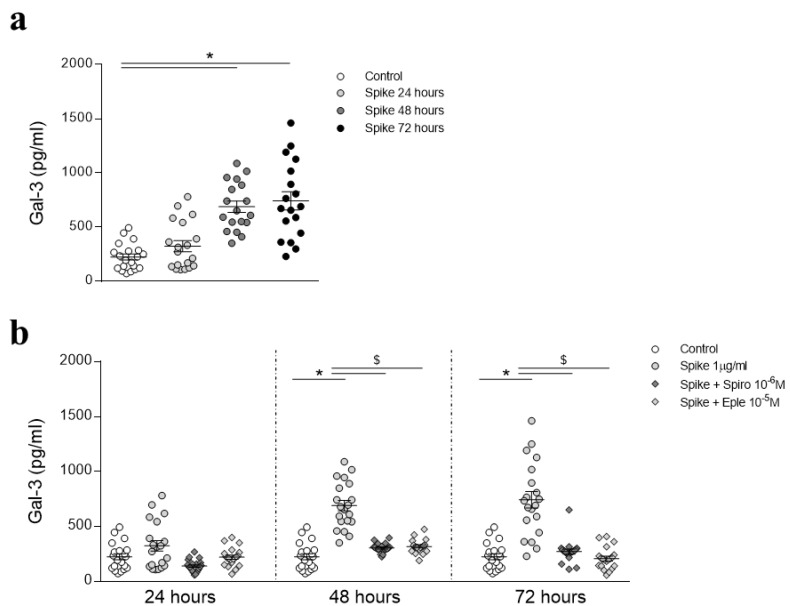
Effects of recombinant Spike protein on Gal-3 secretion in HAECs. The secretion of Gal-3 was evaluated in HAECs treated with recombinant Spike protein for 24, 48, and 72 h (**a**). Gal-3 extracellular levels were measured in HAECs pre-treated with Spironolactone or Eplerenone and then treated with Spike for 24, 48, or 72 h (**b**). Control, *n* = 20; Spike, *n* = 20; Spike + Spiro, *n* = 20; Spike + Eple, *n* = 20. Results were analyzed using one-way-ANOVA followed by Dunnet’s post-hoc multiple comparisons tests. * vs. control; $ vs. Spike.

Interestingly, incubation of HAECs with recombinant Spike significantly induced Gal-3 secretion at 48 (3.15-fold, *p* < 0.0001) and 72 (2.93-fold, *p* < 0.0001) hours (Figure 3a). Moreover, both Spironolactone and Eplerenone blocked Spike-induced Gal-3 at 48 and 72 h (Figure 3b).

### 3.2. Preventive MR/Gal-3 Pathway Inhibition on SARS-CoV-2 Spike Protein-Mediated Inflammatory Effects in HAECs

**Figure 4:**
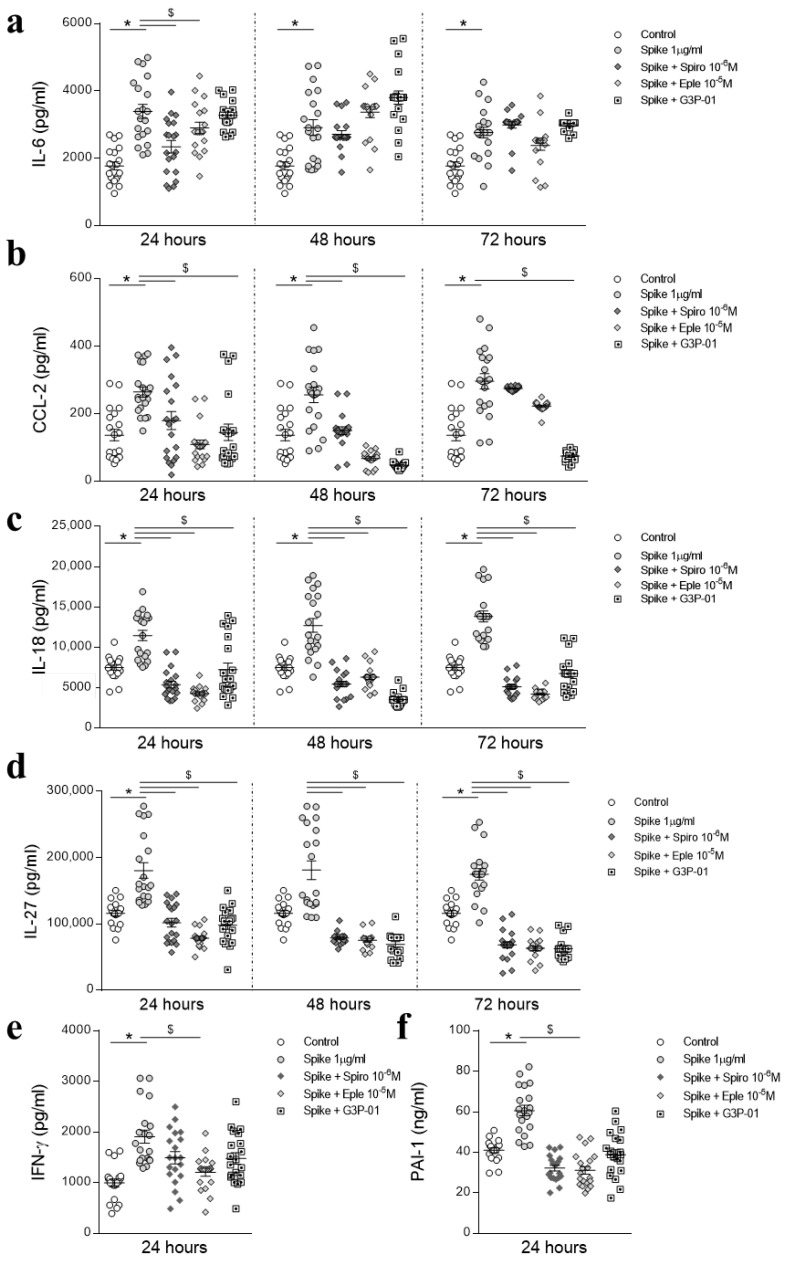
Preventive effects of MR pathway blockade on Spike protein proinflammatory effects in HAECs. The secretion of proinflammatory molecules was evaluated in HAECs pre-treated with Spironolactone, Eplerenone, or G3P-01 and then treated with Spike for 24, 48, or 72 h. IL-6 (**a**), CCL-2 (**b**), IL-18 (**c**), IL-27 (**d**), IFN-γ (**e**), PAI-1 (**f**). Results were analyzed using one-way-ANOVA followed by Dunnet’s post-hoc multiple comparisons tests. Control, *n* = 20; Spike, *n* = 20; Spike + Spiro, *n* = 20; Spike + Eple, *n* = 20; Spike + G3P-01, *n* = 20. * vs. control; $ vs. Spike.

HAECs were pre-incubated with two MRAs with different specificity and efficacy, Spironolactone or Eplerenone, or with the Gal-3 inhibitor G3P-01, and then concomitantly treated with SARS-CoV-2 recombinant Spike for 24, 48, and 72 h. As shown in Figure 4a, MRAs prevented from Spike-increased IL-6 at 24 h but not at 48 or 72 h. Eplerenone and G3P-01 prevented from Spike-induced CCL-2 expression at 24 and 48 h, although only G3P-01 was able to restore normal CCL-2 levels at 72 h (Figure 4b). The increased expression of IL-18 and IL-27 induced by Spike was prevented by both MRAs and G3P-01 at all the timepoints (Figure 4c–d). Only Eplerenone prevented from Spike-increased IFN-γ and PAI-1 at 24 h (Figure 4e–f).

### 3.3. Inhibition of the MR/Gal-3 Pathway on SARS-CoV-2 Spike Protein-Mediated Inflammatory Effects in HAECs

**Figure 5:**
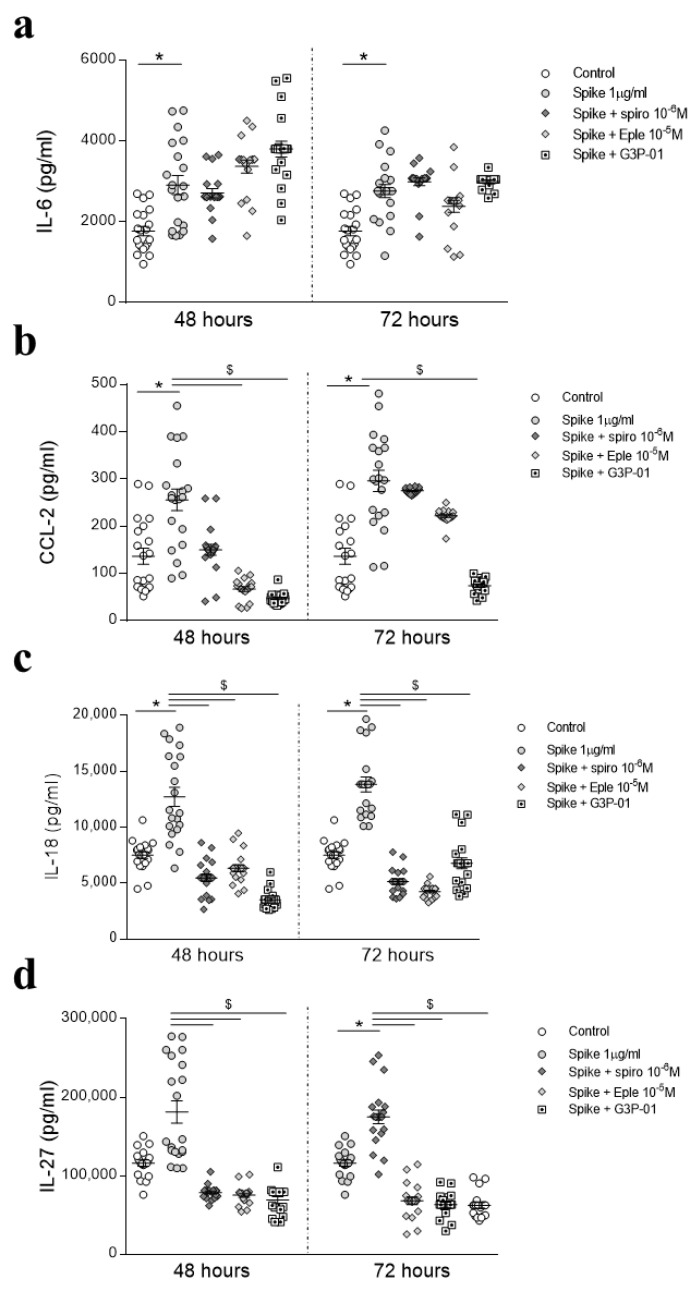
Protective effects of MR pathway blockade after 24 h-Spike protein treatment in HAECs. The secretion of proinflammatory cytokines was evaluated in HAECs treated with recombinant Spike protein for 24 h and then with Spironolactone, Eplerenone, or G3P-01 for 48 or 72 h. IL-6 (**a**), CCL-2 (**b**), IL-18 (**c**), IL-27 (**d**). Control, *n* = 20; Spike, *n* = 20; Spike + Spiro, *n* = 20; Spike + Eple, *n* = 20; Spike + G3P-01, *n* = 20. Results were analyzed using one-way-ANOVA followed by Dunnet’s post-hoc multiple comparisons tests. * vs. control; $ vs. Spike.

HAECs were pre-treated with recombinant SARS-CoV-2 Spike protein for 24 h to mimic an active SARS-CoV-2 infection and then MR antagonists or G3P-01 were added for further 24 or 48 h. As shown in Figure 5a, none of the inhibitors blocked IL-6 increase triggered by pre-treatment with recombinant Spike. CCL-2 upregulation induced by Spike was abolished by Eplerenone and G3P-01 at 48 h and by G3P-01 at 72 h (Figure 5b). The increase in IL-18 and IL-27 induced by pre-treatment with Spike was blocked by MRAs and G3P-01 at all the timepoints (Figure 5c–d).

## 4. Discussion

Recombinant SARS-CoV-2 Spike treatment triggers an acute inflammatory response on HAECs as evidenced by a sustained release of cytokines. MRAs or Gal-3 inhibitors prevented and reversed recombinant SARS-CoV-2 Spike most proinflammatory effects. To our knowledge, this is the first study demonstrating the potential therapeutic effects of MR pathway blockade in the context of endothelial inflammation elicited by Spike protein in endothelial cells.

The immunological and physiological functions, and their systemic distribution, make the endothelial cell an interesting target cell to treat the pathogenesis of COVID-19 [23]. In the present study, SARS-CoV-2 Spike consistently down-regulated ACE2 expression, in agreement with previous publications [24,25]. Accordingly, SARS-CoV-2 infection might diminish ACE2 expression in target organs such as the heart, thus promoting severe heart injury [25]. It has been recently described that SARS-CoV-2 Spike protein impairs endothelial function via downregulation of ACE2 [26]. The authors suggest that although ACE2 reduction would decrease the virus infectivity, a dysregulated renin-angiotensin system due to ACE2 reduction may exacerbate endothelial dysfunction, leading to endothelitis [26]. In line with these findings, treatment with recombinant SARS-CoV-2 spike protein induced the secretion of proinflammatory cytokines in vascular endothelial cells, reinforcing the idea that vascular endothelial cells are active drivers of COVID-19. Moreover, Spike protein increased TMPRSS2 expression in male HAECs. Our results suggest that, in males, this increase may enhance the host priming activity on SARS-CoV-2 spike proteins. In turn, it may result in a higher viral entry. It has been described that TMPRSS2 expression is increased by androgenic hormones, cigarette smoking, type 2 diabetes, H1N1 influenza virus, vanadium pentoxide, or benzo[a]pyrene diol epoxide (Reviewed in [27]). In this regard, treatment strategies to block TMPRSS2 increase have been proposed [28], since TMPRSS2 expression could affect COVID-19 susceptibility and severity.

MRAs have been proposed as potential therapeutic options for COVID-19 [5,7] due to their anti-inflammatory, anti-fibrotic, and anti-androgenic effects [29]. It has been reported that Spironolactone reduces CCL-2, IFN-γ, or IL-6 expressions in inflammatory cells [30,31,32]. Our results, in agreement with these observations, show for the first time that MRAs effectively blocked the up-regulation of inflammatory cytokines induced by Spike treatment in human vascular endothelial cells. Thus, MRAs may be a new option to reduce endothelial cytokine storm in the context of COVID-19 infection.

The expression of Gal-3 is upregulated by MR signaling acting as a downstream effector of the MR pathway [17,18]. Indeed, Gal-3 has already been described to mediate the effects of MR activation in cardiovascular cells [17,18] and has been shown to stimulate inflammation [17,33]. It has been suggested that elevated Gal-3 levels can participate in the cytokine storm reported in patients with severe COVID-19 [34]. It is worth to note that single-cell RNA sequencing analysis has identified Gal-3 to be significantly elevated in bronchoalveolar immune cells in patients with severe COVID-19 compared to mild disease [35]. Moreover, a structural homology has been reported between Spike protein and Gal-3 [36]. Upon ACE2 binding, such ‘Gal-3-like’ domain may be pivotal to the stabilization of the viral adhesion and so it may facilitate the virus entry. Accordingly, Gal-3 inhibition could disrupt the stability of the SARS-CoV2 binding to the host cell and mitigate the entry of SARS-CoV-2 and the inflammatory response [37]. Several publications have demonstrated the anti-inflammatory potential of Gal-3 inhibitors [17,18]. Gal-3 inhibition reduces the release of proinflammatory cytokine from immune cells [38,39]. Our results support that Gal-3 inhibition blocks the proinflammatory response induced by Spike protein and may prevent Spike-induced inflammation, suggesting that Gal-3 blockade could exert dual benefits in the treatment of COVID-19. Moreover, the anti-fibrotic effects of Gal-3 inhibitors may also limit the development of pulmonary fibrosis, most likely a major deleterious consequence in survivors of severe COVID-19.

In brief, our findings suggest that MR/Gal-3 axis is involved on the pathogenesis of COVID-19. Accordingly, pharmacological inhibition of MR pathway could limit the proinflammatory effects induced by SARS-CoV-2 Spike protein in human vascular endothelial cells (see Graphical Abstract). MR and Gal-3 inhibitors might be promising therapeutic options to palliate cytokine inflammatory storm in COVID-19 patients and its long-term clinical impact including pulmonary fibrosis [33,40]. These treatments could add additional protection for COVID-19 infection as per the Spike protein and Gal-3 shared similarities. Future clinical trials are warranted to evaluate the therapeutic potential of MRAs and Gal-3 inhibitors on inflammation and other related effects of MR signaling on blood clotting or fibrosis. Meanwhile, we will remain expectant to the release of results of ongoing clinical trials testing the effect of MRA spironolactone alone (NCT04345887) or combined with other drugs (NCT04278404, NCT04424134, NCT04826822, NCT04643691) on the clinical outcome of different subsets of COVID-19 patients (visit clinicaltrials.gov for more information).

## Figures and Tables

**Table 1:** Major resources table.

Target	Vendor	Catalog #	Working Conc.	Application
ACE2	Abcam	ab15348	20 μg/mL	WB
TMPRSS2	Santa Cruz	sc-515727	2 μg/mL	WB
β-Actin	Santa Cruz	sc-47778	2 μg/mL	WB
IL-6	R&D Systems	DY206	As recommended ^1^	ELISA
CCL-2	R&D Systems	DY279	As recommended ^1^	ELISA
IL-18	R&D Systems	DY318-05	As recommended ^1^	ELISA
IL-27	R&D Systems	DY2526	As recommended ^1^	ELISA
IFN-γ	R&D Systems	DY285B	As recommended ^1^	ELISA
PAI-1	R&D Systems	DY1786	As recommended ^1^	ELISA
Gal-3	R&D Systems	DY1154	As recommended ^1^	ELISA

**#**, Catalogue number; ^1^, as recommended by the manufacturer. ACE2, Angiotensin-converting enzyme 2; TMPRSS2, N-terminal S1 subunit and C terminal S2 subunit by the host transmembrane protease serine 2; IL, interleukin; CCL-2, C-C Motif Chemokine Ligand 2; IFN-γ, Interferon gamma; PAI-1, Plasminogen activator inhibitor-1; Gal-3, Galectin-3; WB, Western blotting; ELISA, Enzyme-Linked Immuno Sorbent Assay.

## Data Availability

The data that support the findings of this study are available from the corresponding author, N.L.-A., upon reasonable request.
